# Editorial: Agile leadership in the light of efficiency of organizations and the health of employees

**DOI:** 10.3389/fpsyg.2023.1294169

**Published:** 2023-10-11

**Authors:** Paul Jimenez, Borut Milfelner, Anita Bregenzer

**Affiliations:** ^1^Institute of Psychology, University of Graz, Graz, Austria; ^2^Faculty of Economics and Business, University of Maribor, Maribor, Slovenia

**Keywords:** agility, innovation, flexible work, new ways of working, organizational culture, leadership culture, working conditions

The digital age is characterized by rapid progress and constantly changing framework conditions and presents companies with unique challenges that can be described by the acronym VUCA (Volatility, Uncertainty, Complexity, and Ambiguity, see Bennett and Lemoine, 2014; Millar et al., 2018). Organizations are increasingly confronted with sudden and unexpected changes and could cultivate agility as a core capability to navigate these challenges successfully. Key points of agile working can be found in work structures, processes, and ways of cooperation and communication (Crocitto and Youssef, 2003). In 2001, an agile manifesto was published that includes the main statements for agile work (Beck et al., 2001; Hohl et al., 2018). According to these statements, the goal of an agile way of working is to be able to constantly improve oneself and one's work by having constant interactions within the team, collaborating with customers, and being flexible to change.

Looking specifically at leadership in agile working, the role of the leader needs to move away from an instructing control authority to a person who can optimally support employees by giving them the appropriate work resources (Bregenzer and Jimenez, 2021). Leaders define and disseminate a guiding vision, which helps employees in making quick decisions and keeps them focused and inspired toward the desired goal (Parker et al., 2015). Agile leaders must be able to create an innovation-friendly environment where employees can act autonomously, and share knowledge and information to help and support each other (Sherehiy and Karwowski, 2014).

The lack of a standardized definition for agile working beyond the software industry poses a challenge when researching this crucial topic. One article in this Research Topic focuses on measuring agility independently of the work sector. The study of Petermann and Zacher identifies 10 dimensions of workforce agility that measure agility from the employees' point of view. These dimensions can help us better to understand the agility in a specific context or can be used to measure workforce agility in a more general term.

In this Research Topic, leadership behavior and its possible relationships with the agile work environment are highlighted in three articles.

During the present time of adversity, empowering employees is one important aspect that leaders should devote themselves to. Leaders that show empowering behavior toward their employees delegate the authority away from the leader back to the team and support their employees in their personal development and decision-making. Ye et al. showed that employees feel supported, attended, and encouraged by having a leader that offers empowering behavior and a stronger sense of belonging and loyalty.

The study of Kao et al. revealed that transformational leadership behavior and a supportive organization play a beneficial role in shaping the employees' altruistic behavior and organizational citizenship behavior. These behaviors are needed in an agile working environment, as self-initiative and commitment are essential for a good agile way of working.

An innovative approach toward how leadership behavior can be classified regarding today's challenges is presented in the article of Stoker et al., where leadership behavior can be categorized in three archetypes. These archetypes vary in terms of the time leaders spend with their employees and the leader's communication style. These two leadership dimensions are of high value in current challenging times.

Three articles on this special topic provide a more nuanced view of how certain leadership styles can have possible critical and beneficial effects on employee outcomes.

According to Chen and Haga, in the concept of differential leadership, leaders show different leadership behaviors within their team depending on how close their employees are to them. The closeness between leader and team member leads to a division between “insiders” and “outsiders.”

In their meta-analysis, Lu et al. explore the relationships between three subdimensions of parental leadership (benevolent leadership, moral leadership, and authoritarian leadership) on the employees' innovation. In today's flexible and challenging work environment, leaders should adjust their weight on each aspect of paternalistic leadership to find the best combination that works in agile teams.

In So's study, the interesting aspect of the manager's overconfidence is investigated. Overconfident managers are overly optimistic and enthusiastic about future projects and prospects and underestimate market risks. In agile organizations, where it is necessary to react quickly and adequately to market changes, this leadership characteristic can have critical effects, therefore, special emphasis on enhancing self-reflection and self-learning among managers should be given.

Contextual factors (e.g., organizational culture, working conditions, diversity) strongly influence leadership behavior. Considering the contextual factors in which leaders can operate holds significant importance in leadership research (Oc, 2018). Four articles deal with the question of how the working environment should be designed to be prepared for today's challenges of the flexible working world.

Weirauch et al. discuss an interesting approach to structuring and managing an organization with the concept of holacracy. In holacratic companies, employees hold roles instead of titles and authority, and possibilities for decision-making are distributed throughout circles, an approach that fits the increasing agile work environment. In such companies, employees feel more appreciated and receive less illegitimate tasks. However, this approach might not fit every person, thus Weirauch et al. stress the importance of a person-organization fit to achieve the best outcomes.

For companies, with a strong focus on customers, where a high level of customer orientation is a priority, agility becomes a crucial resource. In the service sector, where the own emotions have to be regulated constantly and feelings and expressions have to be adjusted to fulfill the customer's needs and wishes, the organization has to give proper support to their employees to be able to deal well with this emotional component. The importance of organizational support especially in the difficult area of the hospitality industry, is highlighted in the study of Hu et al..

Another challenge in today's workplace concerns the issue of temporary employees, where employees are engaged only for a limited period. The study of Xia et al. shows that organizations need to ensure that temporary employees feel accepted and recognized. If that need of belonging is fulfilled, the need to look for another job elsewhere decreases.

Rožman and Milfelner address the important issue of age diversity in organizations. They are studying the relationship between intergenerational leadership and the employee engagement and burnout symptoms in different company sizes. In this study, intergenerational leadership practices have been found to be able to reduce physical burnout, particularly in larger companies.

Finally, one article is devoted to the question of what work attitudes employees should be able to work effectively in today's challenging working world. The study by Rizaie et al. investigated the behavior of employees in hospitals during the COVID-19 outbreak. They contribute to how employee performance can be sustained during these times, especially by exploring the role of organizational citizenship behavior and patriotism.

The articles in this Research Topic provide an in-depth view and give a comprehensive perspective of how the work environment should be designed and of how leaders through promoting agility, should adapt their leadership behavior to allow an innovative, efficient, but also health-promoting work behavior in light of the change of the current working world.

## Author contributions

PJ: Writing—original draft. BM: Writing—original draft. AB: Writing—original draft.
